# Shaoyao decoction alleviates TNBS-induced ulcerative colitis by decreasing inflammation and balancing the homeostasis of Th17/Treg cells

**DOI:** 10.1186/s12906-023-04237-9

**Published:** 2023-11-24

**Authors:** Dongsheng Wu, Yu Zhang, Bo Zou, Yi Lu, Hui Cao

**Affiliations:** 1 Department of Anorectal Surgical, The First Hospital of Hunan University of Chinese Medicine, Changsha, China; 2 Department of Internal Medicine, The First Hospital of Hunan University of Chinese Medicine, Changsha, China

**Keywords:** Shaoyao decoction, Ulcerative colitis, IL-6/STAT3 signaling pathway, Th17/Treg balance

## Abstract

**Background:**

Ulcerative colitis (UC) is a persistent and non-specific inflammatory condition that mainly affects the bowels and has challenging treatment. UC has a growing incidence and significantly affects the well-being of patients. Many medications used to treat UC can disrupt the metabolism and immune system homeostasis, frequently leading to significant adverse effects. Hence, exploring alternative therapies, such as traditional Chinese medicine and probiotics, has recently emerged as a primary research hotspot owing to their safety. Although the therapeutic mechanism of Shaoyao decoction has not been clarified, it has demonstrated a beneficial clinical effect on UC.

**Aim:**

This study aimed to assess the effect of Shaoyao decoction on a rat model of UC and investigate its underlying mechanisms.

**Methods:**

The rat model of UC was induced by 2,4,6-trinitrobenzenesulfonic acid (TNBS). The extent of damage to the intestines was assessed using the disease activity index (DAI), colonic mucosa damage index (CMDI), and histological scores. Immunohistochemistry was employed to detect the tissue levels of interleukin (IL)-17, transforming growth factor (TGF)-β1, and IL-10. Additionally, the proportion of Th17 and Treg cells was detected using flow cytometry. In colon tissue, the levels of forkhead box (Fox)p3, RAR-related orphan receptor (ROR)γt, IL-6, p-STAT3, and STAT3 proteins were quantified by Western blotting.

**Results:**

Treatment with Shaoyao decoction enhanced the overall health of rats and reduced colonic damage. Additionally, Shaoyao decoction significantly alleviated the severity of DAI, CMDI, and HS. The proportion of Th17 cells was reduced, and the proportion of Treg cells was increased by Shaoyao decoction. The expression of IL-17 and RORγt was suppressed by Shaoyao decoction, while the expression of IL-10, TGF-β1, and Foxp3 was increased. The expression of IL-6, p-STAT3, and STAT3 was decreased by Shaoyao decoction.

**Conclusion:**

The Shaoyao decoction alleviates the symptoms of TNBS-induced UC by decreasing inflammation and mitigating intestinal damage while preserving the balance between Th17 and Treg. Shaoyao decoction modulates the IL-6/STAT3 axis, thereby regulating the balance between Th17 and Treg cells.

**Supplementary Information:**

The online version contains supplementary material available at 10.1186/s12906-023-04237-9.

Ulcerative colitis (UC) is the long-lasting and non-specific inflammation of the digestive tract that greatly impacts the overall health of individuals by causing both physical and mental distress [1]. Treating UC is challenging, and its global prevalence is on the rise, which significantly impacts public health [2]. The exact cause of UC is not completely understood, and an imbalanced immune system response is deemed to lead to UC [3]. Th17 (helper T cell 17) and Treg (regulatory T cells), two subpopulations of CD4 + effector T cells, are both critically involved in the pathogenesis of UC. An imbalanced immune response caused by Th17 cells, which stimulate immune response, and Treg cells, which induce immune tolerance, can significantly affect the development of UC [4]. Studies have shown that an increased abundance of Th17 cells in UC can elevate the serum levels of interleukin (IL)-17, whereas a reduced abundance of Treg cells can weaken the anti-inflammatory response [5]. Thus, modulation of Th17/Treg balance, particularly promoting Treg cell response and inhibiting Th17 cell response, may effectively treat UC [6]. The IL-6/STAT3 pathway is closely related to the differentiation of Treg and Th17 cells. Excessive activation of IL-6/STAT3 upregulates RORγt, a transcription factor for Th17 cells, in intestinal naïve T cells and downregulation of Foxp3, a transcription factor for Treg cells, thereby shifting the differentiation of naïve T cells toward Th17 cells and resulting in Treg/Th17 imbalance [7, 8]. Hence, Treg/Th17 imbalance, which is controlled by the IL-6/STAT3 pathway, plays a critical role in the initiation and progression of UC.

Currently, UC is mainly treated by conventional medications such as aminosalicylates, corticosteroids, immunosuppressants, and biological agents [9, 10]. These medications can suppress the inflammatory response. However, their adverse effects in the long term can lower the quality of life [11]. Hence, researchers are increasingly investigating effective substitutes with fewer side effects.

With fewer negative side effects, traditional Chinese medicine (TCM) is a dependable option and supplementary treatment [12]. The Shaoyao decoction is a compound of TCM. In a previous study, it was determined that Shaoyao decoction enhances the anti-inflammatory response through the TLR4/NF-κB pathway in UC [13]. Another study showed that Shaoyao decoction relieves UC by modulating the PI3K/AKT-mTOR and S1P3 signaling pathways [14]. Shaoyao decoction can promote mucosal repair in animal models of UC by inhibiting epithelial apoptosis [15]. Several studies indicated that modulation of the IL-6 signaling pathway is a viable therapeutic approach for UC, as it regulates the balance between Th17 and Treg cells [16, 17]. Few studies examined the effects of Shaoyao decoction on UC by regulating IL-6/STAT3 signaling pathway and Th17/Treg cell balance. Hence, this study aimed to establish an animal model of UC and clarify the relationship between the IL-6/STAT3 pathway and Treg/Th17 cell balance. Additionally, we aimed to investigate the involvement of IL-6/STAT3 signaling pathway in the effect of Shaoyao decoction on Th17/Treg balance. Our ultimate goal was to uncover the mechanism through which Shaoyao decoction treats UC.

## Experimental procedures

### Animals

We purchased 60 male Sprague–Dawley rats from Hunan SJA Laboratory Animal Co, Ltd. (Changsha, China). They aged 5 to 6 weeks and weighed between 150 and 170 g. The animal quality certificate number is 430727211101295026. The rats were kept in conventional cages in a controlled environment free from specific pathogens. The Animal Ethics Committee of the First Affiliated Hospital of Hunan University of Chinese Medicine approved all experimental procedures, with the ethics number ZYFY20210615.

### Reagents

Sigma-Aldrich (Budapest, Hungary) provided 2,4,6-trinitrobenzenesulfonic acid (TNBS). The H&E staining kit was provided by Shanghai Biyuntian Biotechnology Company. Rabbit anti-mouse IL-17, TGF-β1, IL-6, and β-actin antibodies were purchased from Beijing Boaosen Biotechnology. We used sheep anti-rabbit IgG labeled with horseradish peroxidase as the secondary antibody, which was obtained from Beijing Boaosen Biotechnology. The Shaoyao decoction, comprising Radix Paeoniae Alba, Radix Angelicae Sinensis, Rhizoma Coptidis, Semen Arecae, Radix Aucklandiae, Radix Et Rhizoma Glycyrrhizae, Radix Et Rhizoma Rhei, Radix Scutellariae, and Cortex Cinnamomi (Table 1), was purchased from the Outpatient Chinese Medicine Pharmacy at the First Affiliated Hospital of Hunan University of Chinese Medicine. All herbs were from Hunan Sanxiang Traditional Chinese Medicine Pieces Company. After immersing all tablets in distilled water for 2 h, we proceeded with decoction. After filtration, the mixture was concentrated until reaching a drug concentration of 1.1 g/mL. The sustained-release granules of mesalazine were bought from Shanghai Aidifa Pharmaceutical Co. Ltd, with a specification of 0.5 g per bag.

**Table 1:** Details of the ingredients of Shaoyao Decoction

Scientific species names	Family	Weight
*Radix Paeoniae Alba*	Paeoniaceae	30 g
*Radix Angelicae Sinensis*	Umbelliferae	15 g
*Rhizoma Coptidis*	Ranunculaceae	15 g
*Semen Arecae*	Palmae	6 g
*Radix Aucklandiae*	Compositae	6 g
*Radix Et Rhizoma Glycyrrhizae*	Leguminosae	6 g
*Radix Et Rhizoma Rhei*	Polygonaceae	9 g
*Radix Scutellariae*	Labiatae	15 g
*Cortex Cinnamomi*	Lauraceae	5 g

### Model induction and treatment of UC

The rats were randomly divided into six groups. These groups included the control group, TNBS group, MSLQ group (receiving 0.42 g/kg of mesalazine), SYDL group (receiving 11.1 g/kg of low-dose Shaoyao decoction), SYDM group (receiving 22.2 g/kg of medium-dose Shaoyao decoction), and SYDH group (receiving 44.4 g/kg of high-dose Shaoyao decoction). To induce UC, 5 mg TNBS solution was prepared by dissolving it in 0.2 mL of 50% ethanol. All rats except for the control group were given a single intrarectal dose of TNBS after anesthesia with 3% pentobarbital sodium (40 mg/kg). A polyethylene tube with a diameter of 0.4 mm was coated with paraffin oil and introduced into the rectum, reaching a depth of 8 cm. Shaoyao decoction and mesalazine were administered orally for 14 days starting from day 7, while the control group received normal saline in the same period.

### UHPLC LC–MS/MS analysis

We collected the blood samples of the control rats to obtain blank serum. We also collected blood samples from low-dose Shaoyao decoction (11.1 g/kg, SYDL) group. At 4 ֯C, the pure Shaoyao decoction samples, blank serum, and Shaoyao decoction-treated serum were centrifuged at 3000 rpm for 10 min. Next, 200 μL of the supernatant was collected and combined with 800 μL of methanol. The collected supernatant was obtained after centrifuging at a speed of 13,000 revolutions per minute for 10 min. Then, the supernatant was filtered using a microporous membrane with a pore size of 0.22 μm. The filtered samples were utilized for subsequent analyses. The Ultra-high performance liquid chromatography-tandem mass spectrometry (UHPLC LC–MS/MS) analysis was conducted using a Vanquish UHPLC system from Thermo Fisher Scientific, equipped with a Waters UPLC BEH C18 column with 1.7 μm in diameter and 2.1*100 mm in length. The volume of the injected sample was adjusted to 5 µl. A flow rate of 0.5 ml per minute was established. The mobile phase was composed of 0.1% formic acid in aqueous solution (A) and 0.1% formic acid in acetonitrile (B). The program for the multistep linear elution gradient was as follows: from 0 to 11 min, the percentage of A ranged from 85 to 25%; from 11 to 12 min, it ranged from 25 to 2%; from 12 to 14 min, it remained at 2%; from 14 to 14.1 min, it increased from 2 to 85%; from 14.1 to 15 min, it remained at 85%; and from 15 to 16 min, it also remained at 85%. To acquire the MS and MS/MS data using the IDA acquisition mode, the combination of a Q Exactive Focus mass spectrometer and Xcalibur software was utilized. In each acquisition cycle, the mass range varied between 100 and 1500. The top three results of each cycle were selected, and the corresponding MS/MS data were obtained. The flow rate of the sheath gas was 45 Arb, while the auxiliary gas had a flow rate of 15 Arb. The capillary temperature was set at 400 ֯C. The full ms resolution was 70000, and the MS/MS resolution was 17500. In NCE mode, the collision energy was set at 15/30/45. The spray voltage could be either 4.0 kV (positive) or -4.0 kV (negative). During every data acquisition cycle, the molecular ions exhibiting the highest intensity and surpassing 100 were chosen to gather the corresponding secondary MS data. The original mass spectrum was imported using Progenesis QI software. Retention time correction, peak identification, extraction, and integration were performed using MAPS 1.0 BIOTREE software. Biotree's self-constructed secondary mass spectrometry database was used to identify the MS/MS data [18].

### Assessment of intestinal inflammation and DAI

From day 7, we monitored the animals' daily progress in developing experimental colitis using the DAI scoring system [19]. The recorded parameters included body weight loss, stool consistency, and the presence of bloody stool. The DAI score is calculated by dividing the sum of body mass loss score, stool trait score, and stool blood fraction by three. A higher score was associated with more severe inflammation.

### Specimen collection

On the 23rd day, rats in each group were intraperitoneally given pentobarbital to induce anesthesia. Then, they were euthanized by cervical dislocation. Afterward, samples were collected from the colon (from the cecum to the anus) and spleen. The colon was sliced along its longitudinal axis and washed with normal saline. As per Luketal's criteria, the colonic mucosa damage index (CMDI) was evaluated [20].

### Pathological assessment

The colon tissue was fixed in 4% paraformaldehyde solution. Then, it was subjected to paraffin-embedded sections, xylene dewaxing, gradient ethanol solution dehydration, hematoxylin solution staining, 1% hydrochloric acid ethanol fractionation, and eosin solution staining. An expert pathologist, unaware of the study, evaluated the histological score [21]. Part of each colon was taken for other tests.

### Immunohistochemistry

The colon sections were sliced into 4 μm thick sections, then the sections were treated with xylene and ethanol to remove waxes and restore moisture. The sections were treated with a solution of citrate antigen repair, followed by permeabilization by a 0.5% Triton X-100 solution for 20 min. Subsequently, samples were exposed to a 3% H2O2 solution, and then blocked using a 5% bovine serum albumin for 20 min. Thereafter, the sections were incubated with IL-17 (1:500), TGF-β1 (1:500), and IL-10 (1:500) at 37 °C for 1 h. Finally, the sections were incubated with sheep anti-rabbit IgG (1:1 000) at 37 °C for 20 min. The DAB reaction was utilized to visualize the intestinal slides, which were then observed using an optical microscope.

### Flow cytometry for identifying Th17/Treg cells

To obtain individual cell suspensions, we gathered lymphocytes from the spleen and subjected them to centrifugation for 10 min. Initially, the cells were stained using anti-CD4 FITC and anti-CD25-PE-Cy7. Subsequently, they were fixed and permeabilized. Following this, anti-Foxp3 PE or anti-IL-17A PE antibodies were added, and the mixture was incubated at 4 °C for 30 min. Flowjo CE software was used to acquire staining data on a Cytek Dxp Athena flow cytometer. Treg cells were identified as CD4 ^+^CD25^+^ Foxp3^+^ cells, while Th17 cells were identified as CD4^+^ IL-17^+^ cells.

### Western blotting for detecting Foxp3, RORγt, IL-6, p-STAT3, and STAT3

Protease inhibitors and pre-chilled lysate buffer were used to lyse the colon tissues. We subjected the same quantity of protein (50 μg) to 12% SDS-PAGE. Then, the membrane was blocked with Tris-buffered saline Tween 20 buffer that included 5% skim milk. We added primary antibodies (1:1 000 dilution) at 4 °C overnight. The secondary antibodies (1:2000 dilution) were added to the membranes and incubated at room temperature for 1 h. Then, the ECL AB solution was mixed in equal volumes and incubated with the PVDF membrane for 1 min. β-Actin was employed as a control for the sample. Bio-Rad chemiluminescence instrument was used for exposure. We scanned the film with two-color infrared fluorescence scanning instrument, Odyssey. The optical density of the band of interest and the internal reference (β-actin) were determined using Image J software [22].

### Statistical analysis

The SPSS software (ver. 2.0) was utilized to conduct statistical analyses. Data were generated using SPSS (IBM Corp, Armonk, NY, USA), and graphs were designed using GraphPad Prism 8.0 software (San Diego, CA, USA). Continuous variables are expressed as mean ± SD or median (interquartile range [IQR] for continuous variables. For data with normal distribution, one-way ANOVA was utilized to compare variances among three or more groups. The LSD multiple comparison test was employed for comparing the groups. For data without normal distribution, the Kruskal–Wallis test was employed to conduct multiple comparisons, and Dunn's post hoc test was conducted. Statistical significance was determined by considering a P value of less than 0.05.

## Results

### The chemical components of Shaoyao decoction

**Fig. 1:**
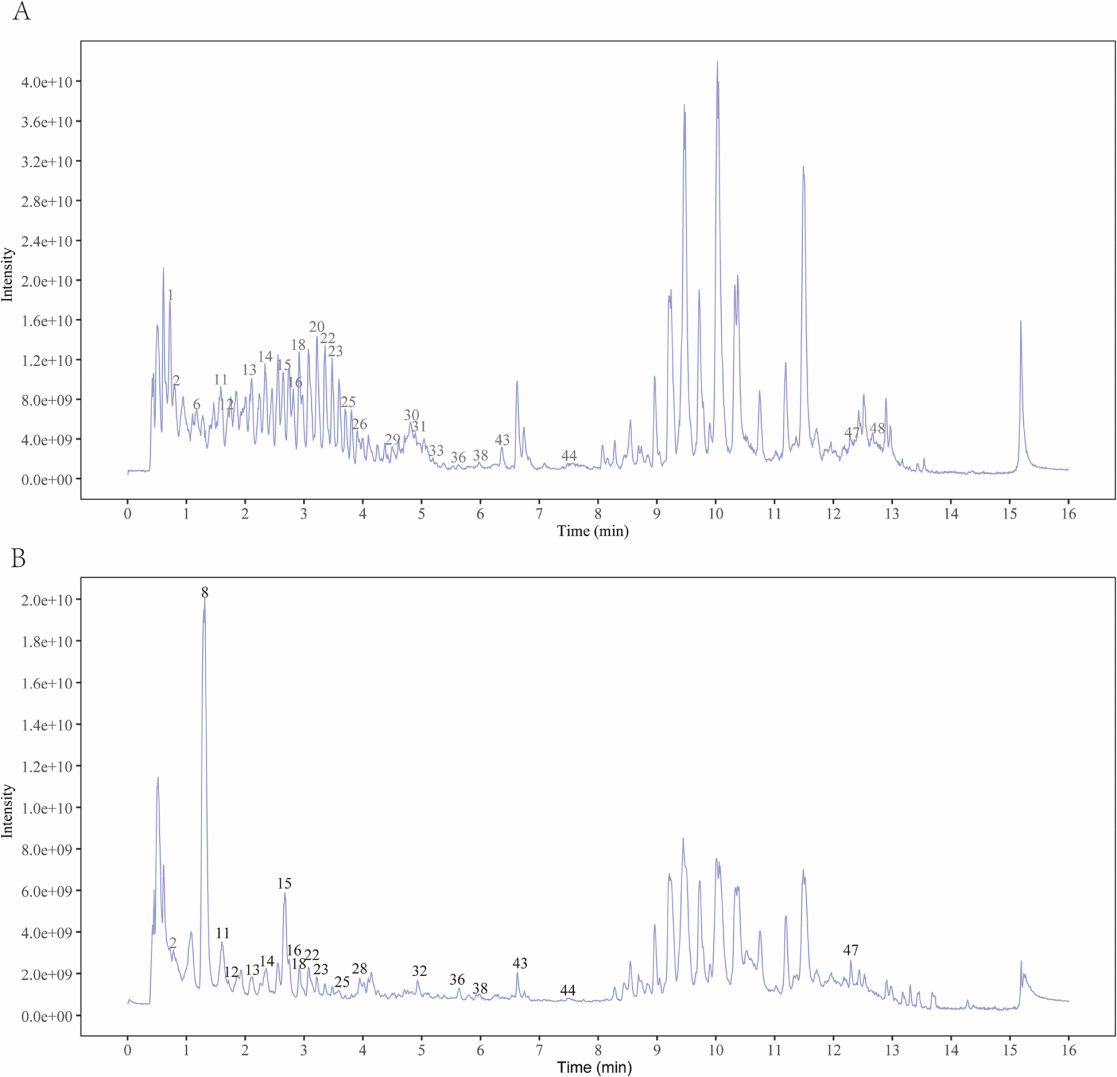
The constituents of Shaoyao decoction in serum were detected By UHPLC LC–MS/MS. **A** Positive ion mode TIC was used to analyze the serum components of Shaoyao decoction. **B** Analysis of serum components of Shaoyao decoction using negative ion mode TIC

Serum samples were analyzed using UHPLC LC–MS/MS to determine the constituents of Shaoyao decoction. Figure 1 displays the LC–MS/MS spectrum of Shaoyao decoction-treated serum in the negative ion/positive ion mode. We successfully detected 49 components of Shaoyao decoction in the bloodstream (Table 2) by analyzing the spectra of pure Shaoyao decoction, untreated serum, and Shaoyao decoction-treated serum.

**Table 2:** The serum components of Shaoyao decoction identified by UHPLC LC–MS/MS

NO	Name	Class	Retention time/min	Chemical formula
1	Caffeoyl quinic acid	phenylpropanoids	0.74079167	C16H18O9
2	( +)-Erythraline	alkaloid	0.78200333	C18H19NO3
3	Quercetin-3-O-glucoside	flavonoids	0.823825	C21H20O12
4	Uracil	Diazines	0.91588333	C4H4N2O2
5	Luteolin-4'-O-glucoside	flavonoids	1.07522	C21H20O11
6	Feruloyl quinic acid	phenylpropanoids	1.14350333	C17H20O9
7	Mearnsitrin	flavonoids	1.20659917	C22H22O12
8	Ellagic acid	phenylpropanoids	1.33214	C14H6O8
9	o-Xylene	Benzene and substituted derivatives	1.36274	C8H10
10	7-methoxy-9,10-dihydrophenanthrene-2,5-diol	Phenanthrenes and derivatives	1.53223333	C15H14O3
11	Scutellarein	flavonoids	1.59420917	C15H10O6
12	Liquiritin	flavonoids	1.69193333	C21H22O9
13	4',5,7-Trihydroxy-6,8-diprenylisoflavone	flavonoids	2.14363333	C25H26O5
14	MORIN	flavonoids	2.333425	C15H10O7
15	Herbacetin	flavonoids	2.64415	C15H10O7
16	Fisetin	flavonoids	2.77781667	C15H10O6
17	Dihydroluteolin	flavonoids	2.78606667	C15H12O6
18	5,7-Dihydroxy-4-methylcoumarin	phenylpropanoids	2.93575	C10H8O4
19	Emodin	quinones	3.16304167	C15H10O5
20	2,3-bis[(4-hydroxy-3-methoxyphenyl)methyl]butane-1,4-diol	phenylpropanoids	3.25765833	C20H26O6
21	Cepharadione B	isoquinoline alkaloid	3.29373333	C19H15NO4
22	N-Acetylanonaine	alkaloid	3.34033333	C19H17NO3
23	Kaempferol 3-O-sulfate	flavonoids	3.4755	C15H10O9S
24	Apigenin	flavonoids	3.54954167	C15H10O5
25	Estragole	Phenol ethers	3.68065833	C10H12O
26	Coumestrol	phenylpropanoids	3.88778333	C15H8O5
27	Sanggenon H	flavonoids	3.91865	C20H18O6
28	Wedelolactone	phenylpropanoids	3.92528333	C16H10O7
29	Hexamethylquercetagetin	flavonoids	4.53515	C21H22O8
30	Flavone base + 3O, O-HexA	flavonoids	4.77920833	C21H18O11
31	Isoliquiritigenin	flavonoids	4.863	C15H12O4
32	1,2-Dihydroxy anthraquinone	quinones	4.86436667	C14H8O4
33	Silybin	flavonoids	5.24036667	C25H22O10
34	Jaceosidin	flavonoids	5.47153333	C17H14O7
35	Licoflavanone	flavonoids	5.47176667	C20H20O5
36	Berlambine	alkaloid	5.62416667	C20H17NO5
37	calycosin	flavonoids	5.82869167	C16H12O5
38	6-Oxochelerythrine	alkaloid	6.04055	C21H17NO5
39	Pinocembrin	flavonoids	6.20543333	C15H12O4
40	(1alpha,6alpha,7alphaH)-2,4(15)-Copadiene	terpenoids	6.452675	C15H22
41	Flavone base + 40, 1Prenyl	flavonoids	6.46931667	C20H18O6
42	Licoricesaponin K2	terpenoids	6.603075	C42H62O16
43	Phloretin	flavonoids	6.68948333	C15H14O5
44	Carpachromene	flavonoids	7.476025	C20H16O5
45	Isoglycyrol	phenylpropanoids	7.50083333	C21H18O6
46	Tri-N-butyl phosphate; CE30; STCOOQWBFONSKY-UHFFFAOYSA-N	Organic phosphoric acids and derivatives	9.11573333	C12H27O4P
47	Alnustone	Diarylheptanoids	12.3221667	C19H18O
48	Withanolide A	terpenoids	12.6997	C28H38O6
49	Terfenadine	alkaloid	13.832	C32H41NO2

### Effects of Shaoyao decoction on general conditions and macroscopic morphology of colon in UC rats

**Fig. 2:**
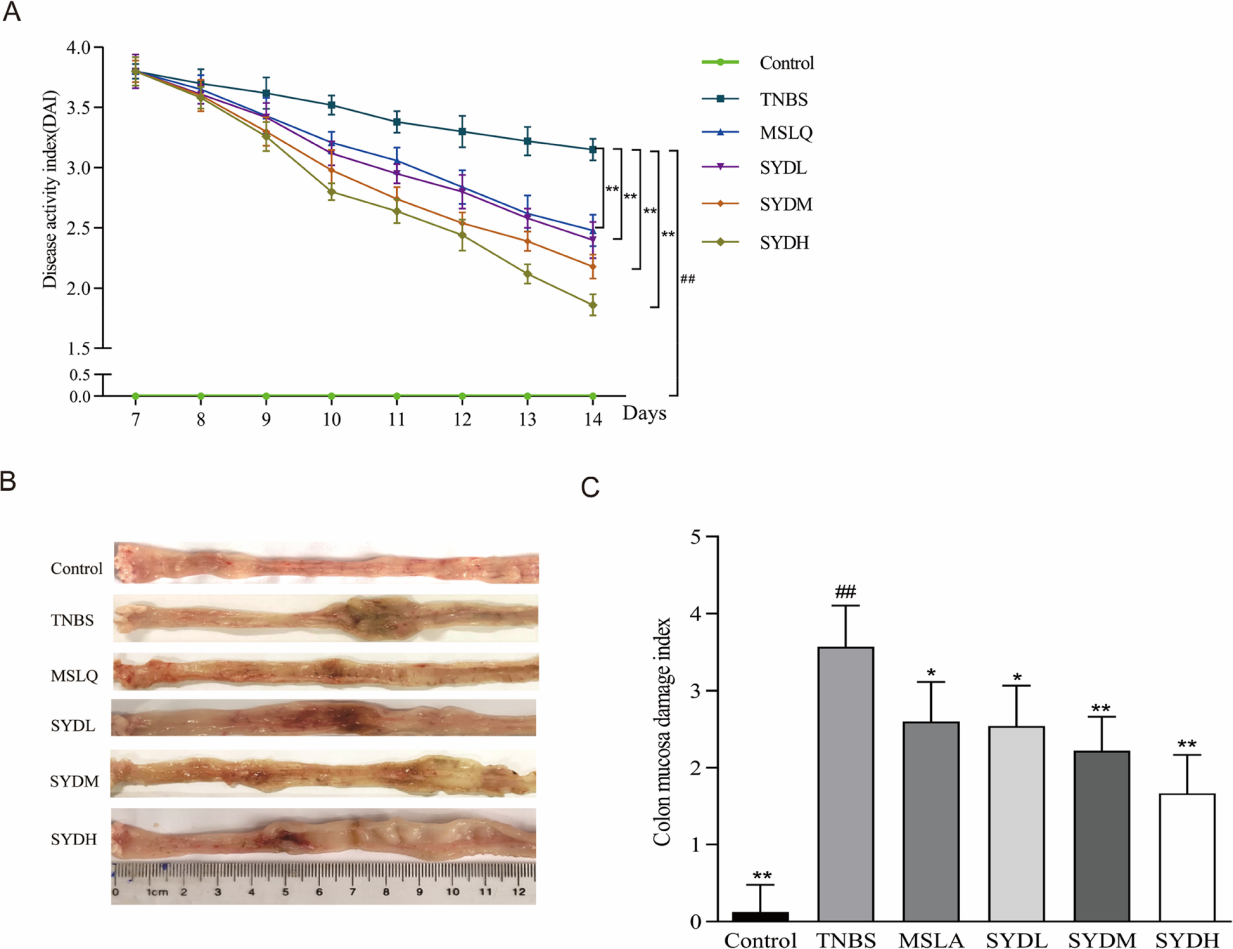
Shaoyao decoction alleviated TNBS-induced UC in rats. **A** DAI evaluation. **B** macroscopic morphology of the colon. **C** CMDI evaluation. Control refers to the control group; TNBS refers to the TNBS group; SYDL refers to the low-dose Shaoyao decoction group; SYDM refers to the moderate-dose Shaoyao decoction group; SYDH refers to the high-dose Shaoyao decoction group. All data are presented as mean ± SD. #*P* < 0.05 and ##*P* < 0.01 compared to the control group; *P < 0.05 and **P < 0.01 compared to the TNBS group. Analysis of variance (ANOVA) conducted in one direction

Initially, we assessed the effectiveness of Shaoyao decoction in relieving the overall health and macroscopic morphology of the colon in rats with UC. Rats in the TNBS group exhibited lackluster fur, mucous-filled stools, and bloody stools, with markedly increased DAI score (Fig. 2A). The DAI scores of the MSLA, SYDL, SYDM, and SYDH groups were lower than that of the TNBS group. In Fig. 2B, the appearance of the colon indicated congestion, edema, erosion, ulceration, and dilation in the TNBS group. Additionally, the CMDI score of rats in the TNBS group was notably increased (Fig. 2C). Rats in all treatment groups exhibited decreased colonic congestion, edema, erosion, ulceration, and dilation compared to the TNBS group. Additionally, the CMDI scores of rats in all treatment groups were reduced. These results showed that Shaoyao decoction improved overall the health of TNBS-induced UC and reduced colonic damage.

### The effects of Shaoyao decoction on colonic inflammation in rats with UC

**Fig. 3:**
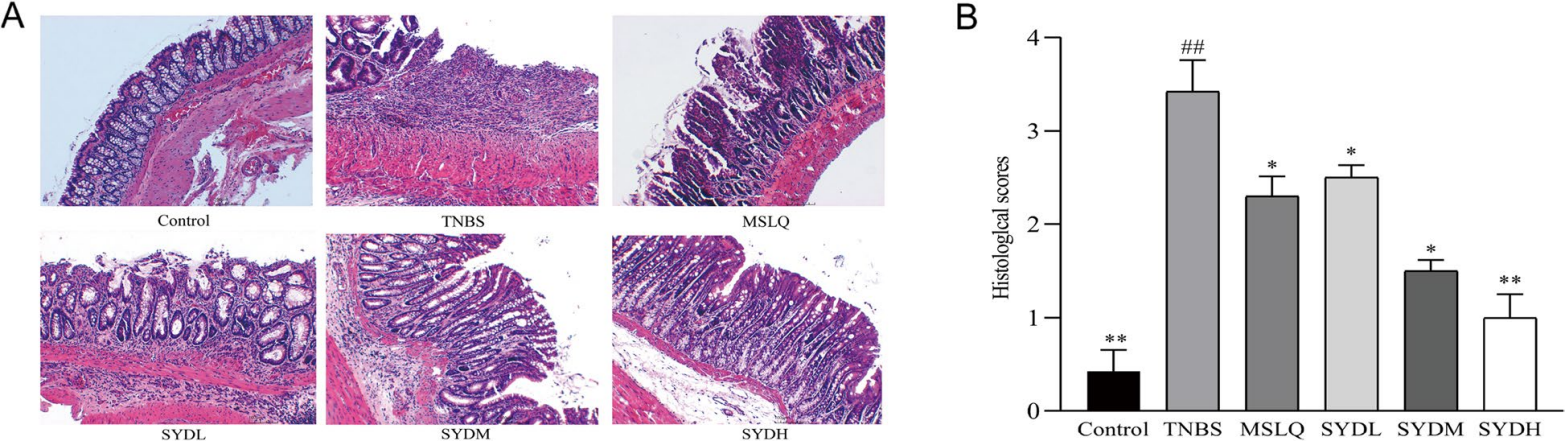
Shaoyao decoction alleviated colon inflammation of UC in rats. **A** Colon tissue was stained with H&E at a magnification of 100x. **B** Histological score. Control refers to the control group; TNBS refers to the TNBS group; SYDL refers to the low-dose Shaoyao decoction group; SYDM refers to the moderate-dose Shaoyao decoction group; SYDH refers to the high-dose Shaoyao decoction group. All data are presented as mean ± SD. #*P* < 0.05 and ##*P* < 0.01 compared to the control group; *P < 0.05 and **P < 0.01 compared to the TNBS group. Analysis of variance (ANOVA) conducted in one direction

The control group had intact colonic structures, with normal crypt and gland structures (Fig. 3A). In the TNBS group, the colon tissue exhibited notable hyperemia and edema, and tissue infiltration of a high abundance of inflammatory cells. The crypts and glands were arranged in a chaotic manner, and the histological score was significantly increased (Fig. 3B). Following treatment with mesalazine and Shaoyao decoction, colonic congestion, edema, and tissue abundance of inflammatory cells were decreased. The intestinal mucosal ulcers healed, and the histological score significantly decreased. Treatment with Shaoyao decoction mitigated the histological alterations in the colon of the TNBS-induced UC.

### The impact of Shaoyao decoction on the protein expression of IL-17, TGF-β1, and IL-10 in the colon tissue of rats with UC

**Fig. 4:**
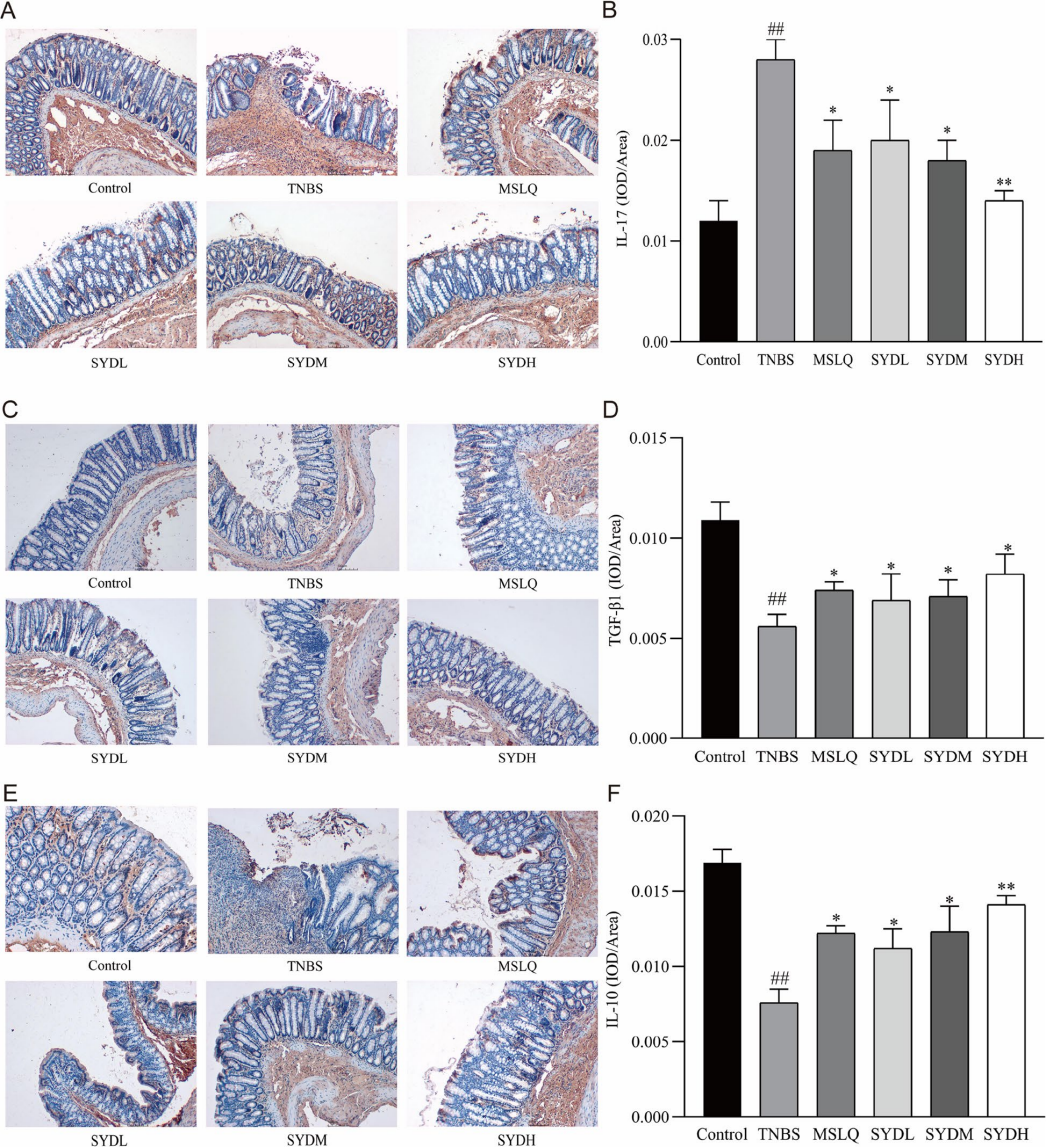
The impact of Shaoyao decoction on the immunohistochemical staining of IL-17, TGF-β1, and IL-10 in the colon tissues of rats with UC (× 100). **A** The immunohistochemical staining of IL-17. **B** Positive expression of IL-17. **C** Representative images showing immunohistochemical staining of TGF-β1. **D** Positive expression of TGF-β1. **E** Representative images showing the immunohistochemical staining of IL-10. **F** Positive expression of IL-10. Control refers to the control group; TNBS refers to the TNBS group; SYDL refers to the low-dose Shaoyao decoction group; SYDM refers to the moderate-dose Shaoyao decoction group; SYDH refers to the high-dose Shaoyao decoction group. All data are presented as mean ± SD. #*P* < 0.05 and ##*P* < 0.01 compared to the control group; *P < 0.05 and **P < 0.01 compared to the TNBS group. Analysis of variance (ANOVA) conducted in one direction

Compared to the control group, the tissue levels of IL-17 were significantly increased in the TNBS group, while tissue levels of TGF-β1 and IL-10 significantly decreased (Fig. 4). On the other hand, the combination of mesalazine and Shaoyao decoction markedly decreased the tissue levels of IL-17 while increasing the tissue levels of TGF-β1 and IL-10 compared to the TNBS group. The results indicated that Shaoyao decoction regulated the release of cytokines associated with Th17 and Treg cells, thereby ameliorating TNBS-induced colonic inflammation.

### The effect of Shaoyao decoction on Th17/Treg ratio in the spleen of rats with UC

**Fig. 5:**
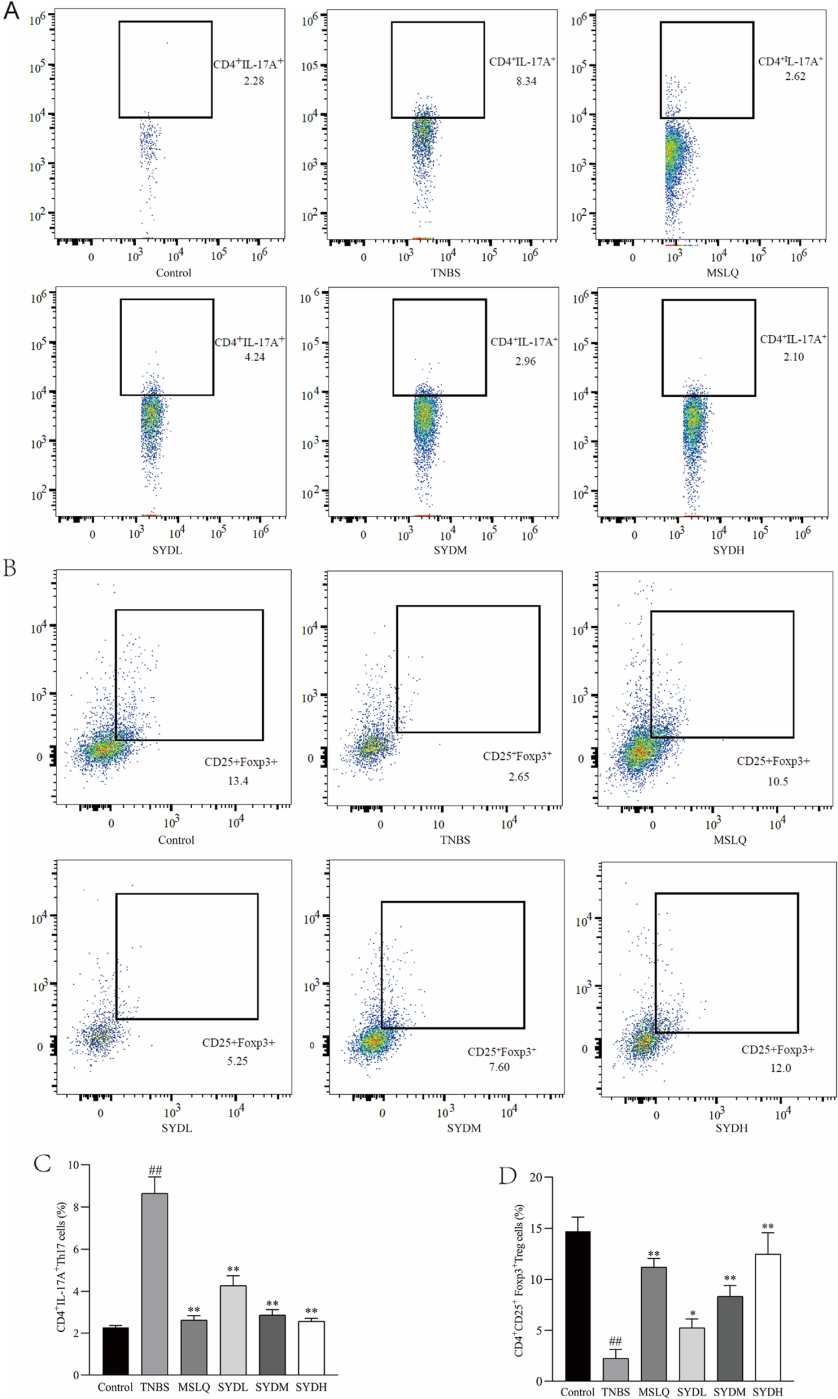
Shaoyao decoction improved the balance between Treg cells and Th17 cells in the colon of rats with UC. **A** The relative abundance of Th17 cells. ** B** Treg cell ratio. **C** Alterations in Th17 lymphocyte ratio. **D** Variations in Treg cell ratio. Control refers to the control group; TNBS refers to the TNBS group; SYDL refers to the low-dose Shaoyao decoction group; SYDM refers to the moderate-dose Shaoyao decoction group; SYDH refers to the high-dose Shaoyao decoction group. All data are presented as mean ± SD. #*P* < 0.05 and ##*P* < 0.01 compared to the control group; *P < 0.05 and **P < 0.01 compared to the TNBS group. Analysis of variance (ANOVA) conducted in one direction

**Fig. 6:**
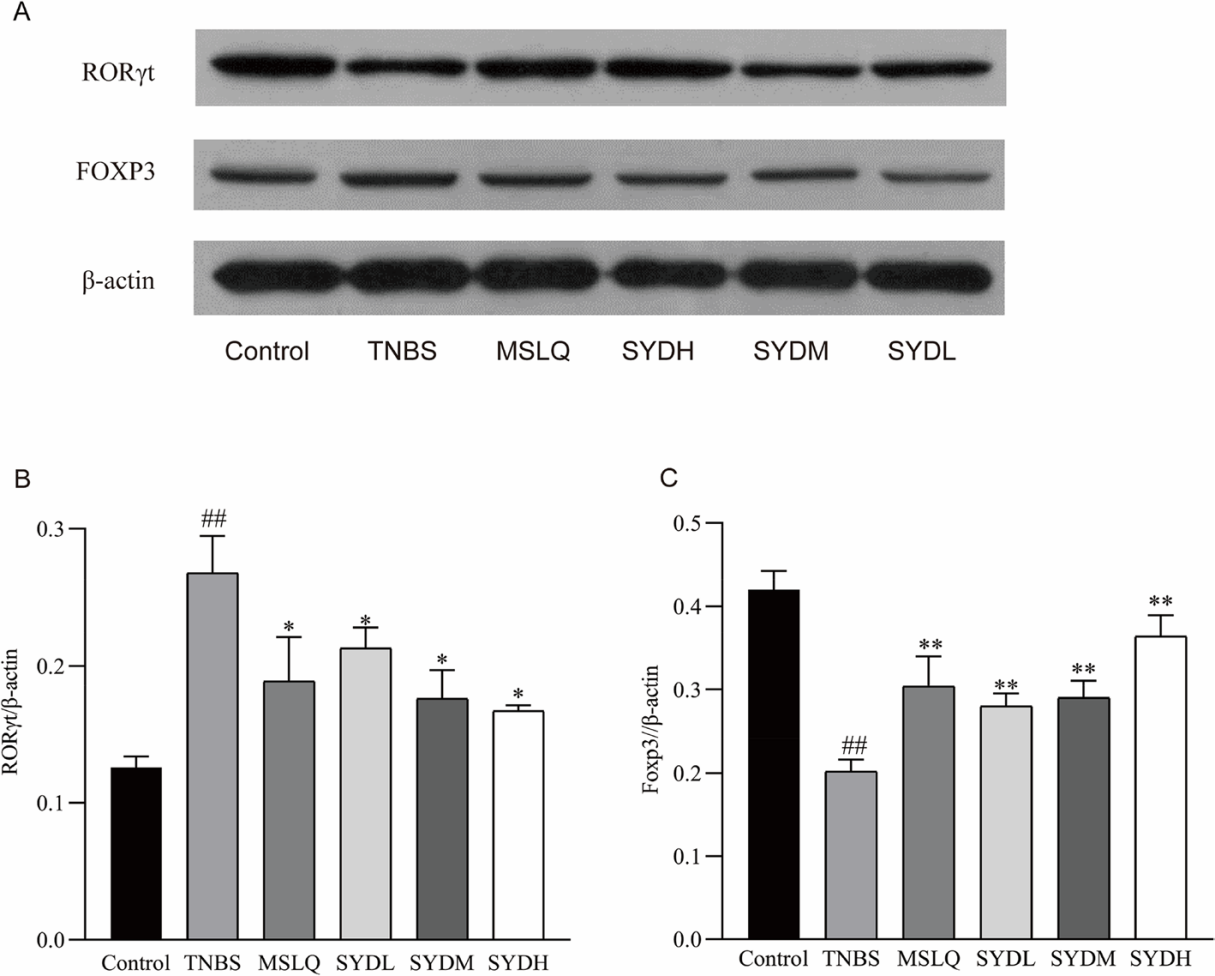
The impact of Shaoyao decoction on the Protein expression of RORγt and Foxp3 in the colon of rats with UC. **A** The impact of Shaoyao decoction on the tissue levels of Th17 and Treg cell-related proteins. **B** The protein levels of RORγt in colon tissue relative to other proteins. **C** The protein levels of Foxp3 in colon tissue relative to other proteins. Control refers to the control group; TNBS refers to the TNBS group; SYDL refers to the low-dose Shaoyao decoction group; SYDM refers to the moderate-dose Shaoyao decoction group; SYDH refers to the high-dose Shaoyao decoction group. All data are presented as mean ± SD. #*P* < 0.05 and ##*P* < 0.01 compared to the control group; *P < 0.05 and **P < 0.01 compared to the TNBS group. Analysis of variance (ANOVA) conducted in one direction

Compared to the control group, the TNBS group had an increased proportion of CD4 + IL-17 + (Th17)/CD4 + lymphocytes (Fig. 5A, B) and decreased proportion of CD4 + Foxp3 + (Treg)/CD4 + lymphocytes (Fig. 5C, D). Compared to the TNBS group, the SYDL, SYDM, and SYDH groups had decreased ratios of CD4 + IL-17 + (Th17)/CD4 + lymphocytes, while increased ratios of CD4 + Foxp3 + (Treg)/CD4 + lymphocytes. We examined how Shaoyao decoction influenced the tissue levels of Th17 and Treg cell-related proteins in the colon tissue of rats with UC. In the TNBS group, Western blotting (Fig. 6A) revealed a significant decrease in Foxp3 expression (Fig. 6B) and a significant increase in RORγt expression (Fig. 6C) compared to the control group. On the other hand, compared to the TNBS group, MSLQ, SYDL, SYDM, and SYDH groups had increased colonic expression of Foxp3 and decreased expression of RORγt. After receiving Shaoyao decoction, the tissue abundance of Treg and Th17 cells returned to normal levels. The results indicated that the Shaoyao decoction reversed the excessive response of the immune system in UC by improving the balance between Th17 cells and Treg cells.

### The impact of Shaoyao decoction on the expression of IL-6, p-STAT3, and STAT3 proteins in the colon tissue of rats with UC

**Fig. 7:**
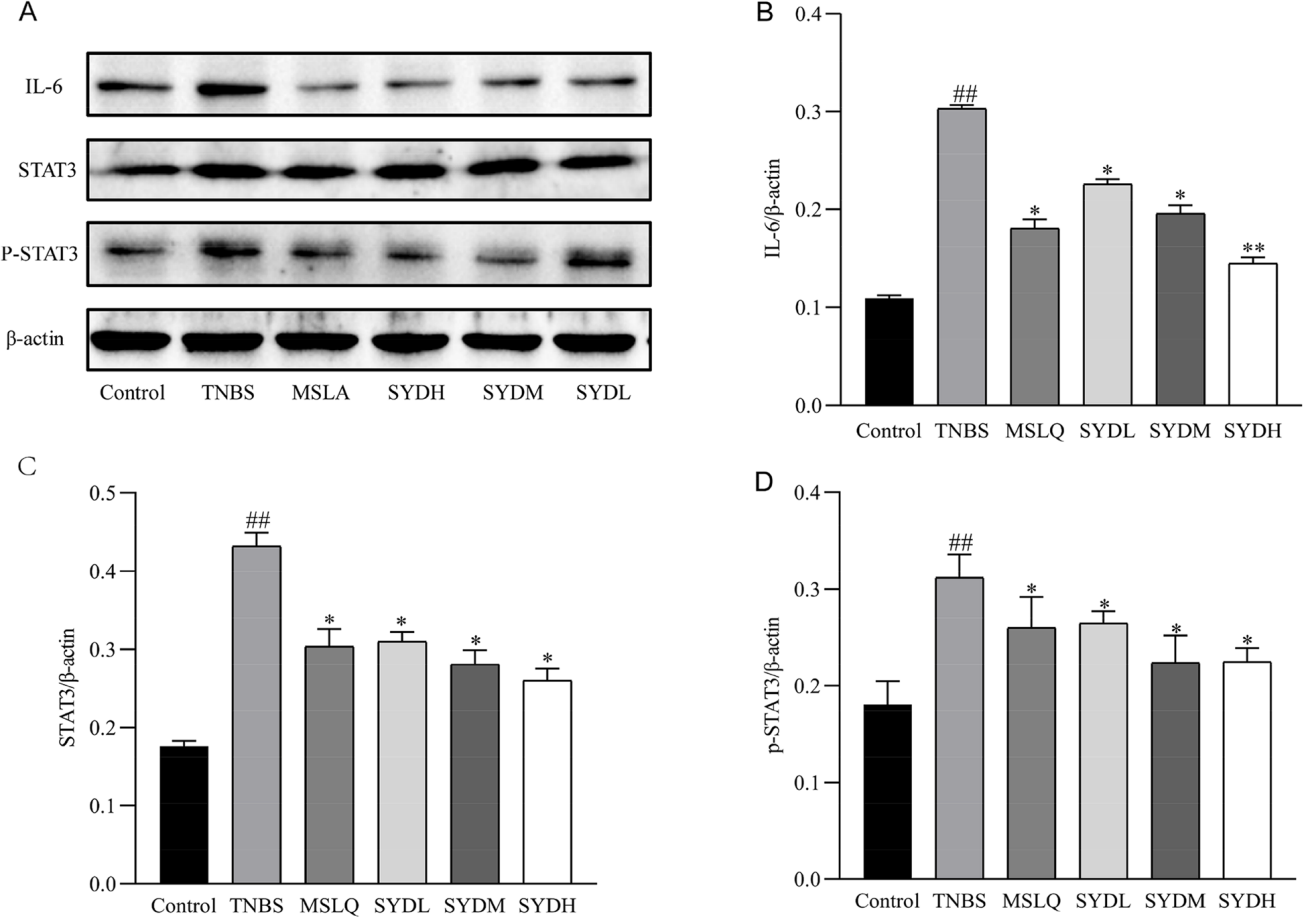
The tissue levels of IL-6, STAT3, and p-STAT3 in the colon mucosa were reduced after treatment with Shaoyao decoction. **A** The protein levels of IL-6, STAT3, and p-STAT3 in the colon tissue were detected using Western blotting. **B** The protein levels of IL-6 in colon tissue compared to other proteins. **C** The protein levels of STAT3 in the colon tissue relative to other proteins. **D** The protein levels of phosphorylated STAT3 in the colon tissue compared to other proteins. Control refers to the control group; TNBS refers to the TNBS group; SYDL refers to the low-dose Shaoyao decoction group; SYDM refers to the moderate-dose Shaoyao decoction group; SYDH refers to the high-dose Shaoyao decoction group. All data are presented as mean ± SD. #*P* < 0.05 and ##*P* < 0.01 compared to the control group; *P < 0.05 and **P < 0.01 compared to the TNBS group. Analysis of variance (ANOVA) conducted in one direction

Compared to the control group, the TNBS group exhibited an increased expression of IL-6, p-STAT3, and STAT3 in the colon tissue (Fig. 7). After administering Shaoyao decoction, the tissue levels of IL-6, p-STAT3, and STAT3 were reduced compared to the TNBS group. Our findings indicated that Shaoyao decoction reduced the tissue levels of IL-6, p-STAT3, and STAT3 in the intestines, thereby modulating intestinal immune function.

## Discussion

Imbalanced immune response in UC is caused by impaired intestinal immune response and endangers the integrity of the intestinal mucosal barrier. Additionally, Th17/Treg imbalance is closely associated with impaired immune response in UC [23–25]. The primary function of Treg cells is to control the behavior of immune cells by releasing anti-inflammatory cytokines like TGF-β and IL-10. These cytokines inhibit the inflammatory response and induce immune tolerance in the peripheral immune system [26, 27]. Th17 and Treg cells utilize a shared signaling pathway through TGF-β [28, 29]. TGF-β activates the STAT3 pathway and promotes Th17 cell differentiation. Furthermore, TGF-β activates the STAT3 pathway in Treg cells through paracrine signaling, thereby recruiting Treg cells and inducing immune tolerance [30]. Shaoyao decoction is widely used as an adjuvant treatment for UC. Shaoyao decoction can alleviate UC by inhibiting NLRP3 inflammasome and NF-κB pathway [31]. Shaoyao decoction protects against intestinal ulcers by inhibiting the MKP1/NF-κB/NLRP3 pathway-mediated pyrosis [32]. To assess the effect of Shaoyao decoction on UC, various indicators of disease severity, including bloody stool, diarrhea, DAI scores, colonic morphology of the colon, histocytokine levels, histopathology, and other parameters, were assessed. Our study showed that UC rats had significantly poor health conditions and decreased body weights. Additionally, there was a significant increase in both DAI and CMDI scores, indicating varying degrees of colonic damage. Treatment with Shaoyao decoction improved the overall health and body weight of rats with UC to different extents, decreased the DAI and CMDI scores, and ameliorated the macroscopic and microscopic alterations of the colon. These findings indicate that Shaoyao decoction can improve the morphological alterations and pathological damages in rats with UC. In addition, TNBS-induced UC increased the expression of IL-17 in the colon, whereas it decreased the expression of TGF-β1 and IL-10. Treatment with Shaoyao decoction notably reduced IL-17 expression in the colon of rats, whereas it elevated the expressions of TGF-β1 and IL-10. These findings imply that Shaoyao decoction modulated the immune response by regulating the expression of IL-17, TGF-β1, and IL-10. We conducted three interventions using different amounts of Shaoyao decoction to treat UC. We found that higher doses of Shaoyao decoction were more effective in reducing colonic pathological damage, DAI scores, CMDI scores, and IL-17 expression in UC rats compared to low and moderate doses. Additionally, higher doses of Shaoyao decoction also more effectively upregulated TGF-β1 and IL-10 expression. These findings indicate that Shaoyao decoction dose-dependently improved UC.

UC causes intestinal inflammation and disrupts the balance between pro-inflammatory and anti-inflammatory responses, overactivating Th17 cells and inhibiting Treg cell response in UC [33]. Maintaining intestinal immune function necessitates the balance between Th17 and Treg cells. Thl7 cells originate from naive T cells. The development and function of Th17 cells are primarily regulated through the expression RORγt [34]. UC is closely linked to Th17 cell-mediated inflammatory response. Treg cells, a subset of T lymphocytes with adverse immunomodulatory effects, are controlled by the transcription factor FOXP3. These cells originate from naive T cells and primarily release inhibitory cytokines, such as IL-10 [35]. Foxp3 + Tregs are present in the mucosal tissue of the colon and inhibit excessive inflammatory response by producing anti-inflammatory cytokines like IL-35, TGF-β, and IL-10 [36]. The expression of IL-10 is reduced in individuals with UC and worsens disease severity due to the impaired function of Foxp3 + Tregs [37]. Foxp3 preserves Treg cell development and confines inflammation. The impact of Shaoyao decoction on the quantity and performance of Th17 and Treg cells was investigated in this study. It was discovered that treatment with Shaoyao decoction notably reduced the abundance of Th17 cells and downregulated the expression of RORγt. Conversely, there was a significant increase in the proportion of Treg cells and in the expression of Foxp3. These findings indicate that Shaoyao decoction protected against UC by modulating Treg/Th17 cell imbalance.

Clinical studies demonstrated a notable increase in IL-6 expression and STAT3 phosphorylation in lamina propria lymphocytes and colonic epithelial cells [38]. Recent studies reported that the IL-6/STAT3 pathway plays a crucial role in T cell differentiation into Treg and Th17 cells [39]. Treg/Th17 imbalance caused by the IL-6/STAT3 pathway is a significant pathological process in the development of UC. Hence, like JAK inhibitors, inhibition of IL-6/STAT3 pathway may treat UC. RORγt activates the IL-6/STAT3 pathway in intestinal naïve T cells, promotes naïve T cell differentiation into Th17 cells, and leads to Treg/Th17 cell imbalance [40]. The expression of IL-6, p-STAT3, and STAT3 in the colon tissue of rats in the TNBS group was found to be markedly higher compared to the control group. Compared to the TNBS group, Shaoyao decoction significantly reduced the expression of IL-6, p-STAT3, and STAT3. Additionally, it significantly decreased the abundance of Th17 cells and enhanced the abundance of Treg cells in the spleen of UC rats. These findings suggest that Shaoyao decoction can regulate the IL-6/STAT3 pathway, thereby modulating Treg/Th17 Treg balance.

The protective mechanisms of Shaoyao decoction may be related to its numerous active components. Shaoyao Decoction analysis by UHPLC LC–MS/MS revealed 49 blood constituents. Certain monomers have been demonstrated to possess protective properties in UC. Rhizoma Coptidis-derived berberine has been utilized for treating UC by modulating gut microbiota and regulating Treg/Th17 ratio [41]. Treatment with total glucosides of paeony decreased the abundance of Th17 cells while increasing the abundance of Treg cells in both spleen and MLN. These findings indicate that total glucosides of paeony improve the balance between Th17 and Treg cells, thereby improving TNBS-induced colitis [42]. Regulating Treg/Th17 balance and modulating gut microbiota and SCFAs by Baicalin from Radix Scutellariae may protect against UC in rats [43].

Collectively, our findings demonstrate that the Shaoyao decoction not only ameliorates the overall health in UC but also suppresses IL-6/STAT3 signaling pathway and improves Treg/Th17 imbalance. Hence, we hypothesized that Shaoyao decoction can protect against intestinal ulcers by modulation Th17/Treg imbalance through the IL-6/STAT3 signaling pathway.

## Conclusion

Shaoyao decoction relieved the symptoms of UC, reduced intestinal inflammation and pathological damage, and maintained Treg/Th17 balance in TNBS-induced UC. The effect of Shaoyao decoction on Th17/Treg balance in UC was associated with the modulation of IL-6/STAT3 signaling pathway. Hence, we hypothesized that Shaoyao decoction improved Th17/Treg balance in rats with UC by modulating the IL-6/STAT3 signaling pathway.

### Supplementary Information

**Additional file 1: Figure 4E.** Loading order: control group, TNBS group, mesalazine group, SYDH group, SYDM group, SYDL group. **Figure 5A.** Loading order in the red circle: control group, TNBS group, mesalazine group, SYDH group, SYDM group, SYDL group.

## Data Availability

The datasets used in the current study are available from the corresponding authors upon reasonable request.
