# Supplementary material for: Shaoyao decoction alleviates TNBS-induced ulcerative colitis by decreasing inflammation and balancing the homeostasis of Th17/Treg cells
Source: BMC Complement Med Ther. 2023 Nov 24;23:424. doi: 10.1186/s12906-023-04237-9 (PMC10668496; doi:10.1186/s12906-023-04237-9)

**Figure 4E**

**
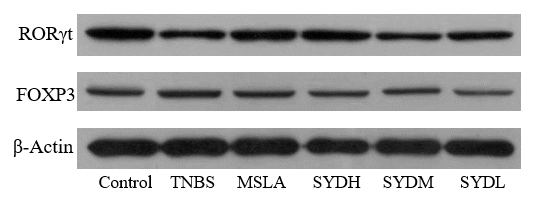
**

**Loading order:** control group, TNBS group, mesalazine group, SYDH group, SYDM group, SYDL group.

ROR-γt（54kDa）

FOXP3（47kDa）


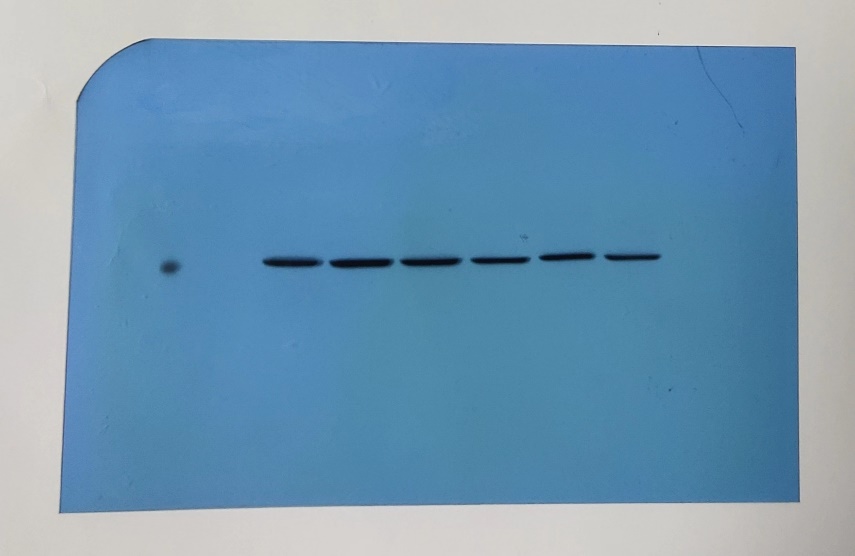
**
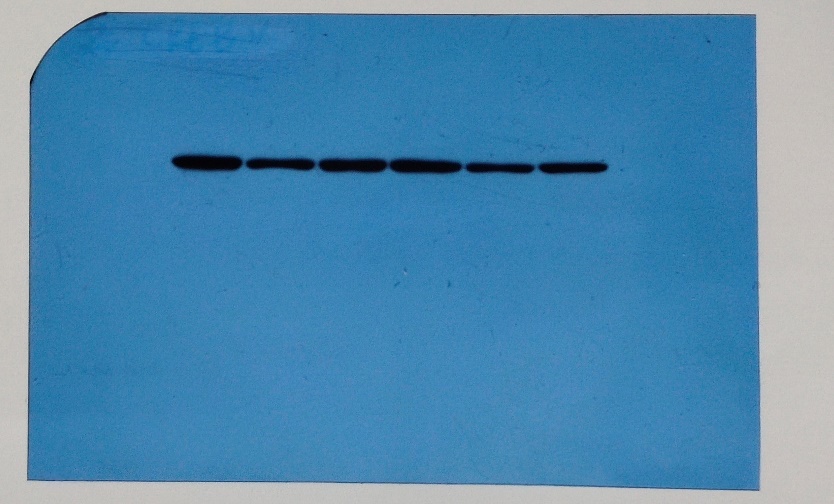
**

β-Actin（42kDa）


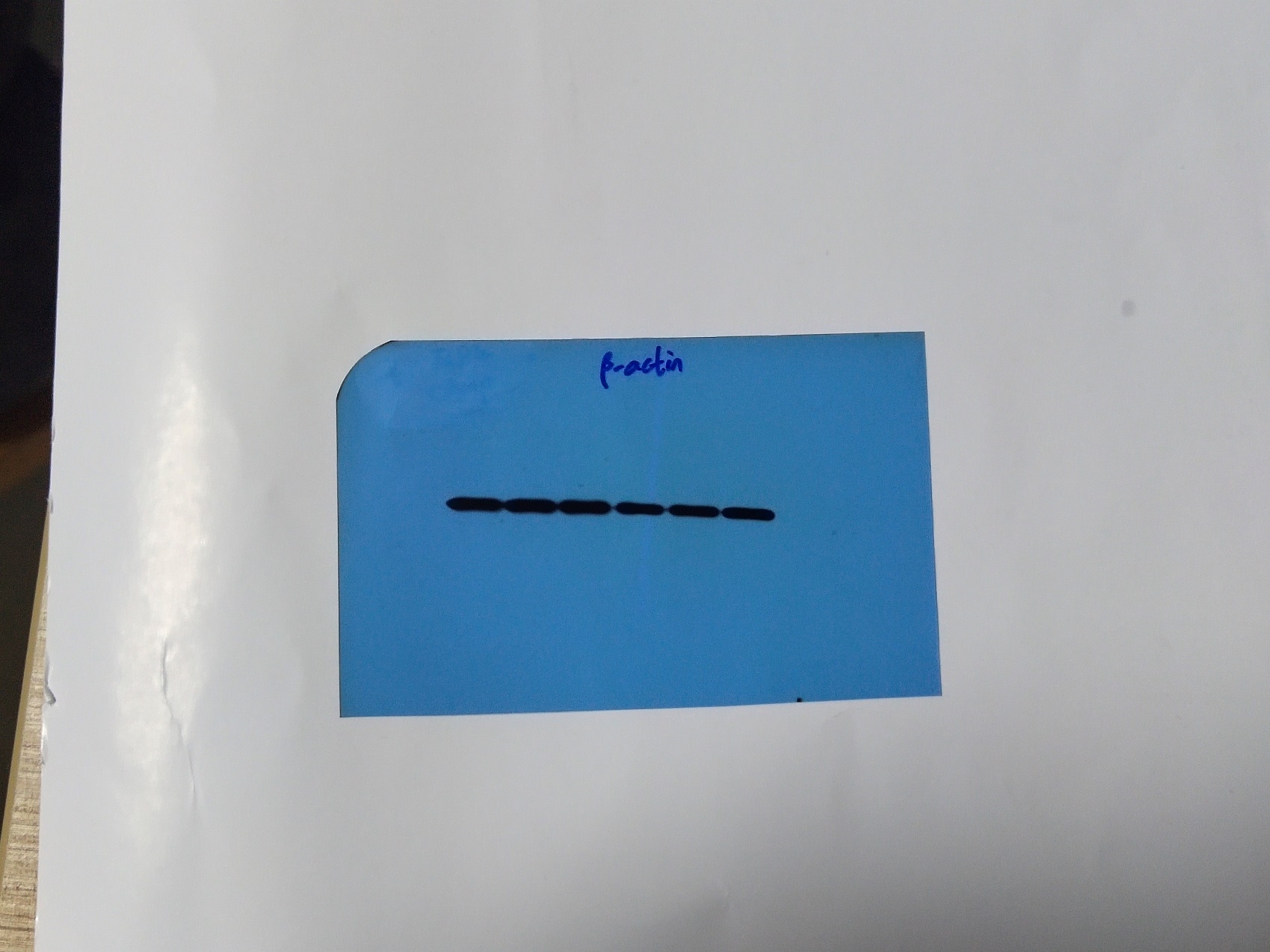


**Figure 5A**

**
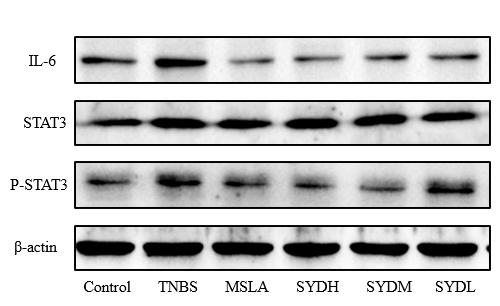
**

**Loading order in the red circle:** control group, TNBS group, mesalazine group, SYDH group, SYDM group, SYDL group.

**
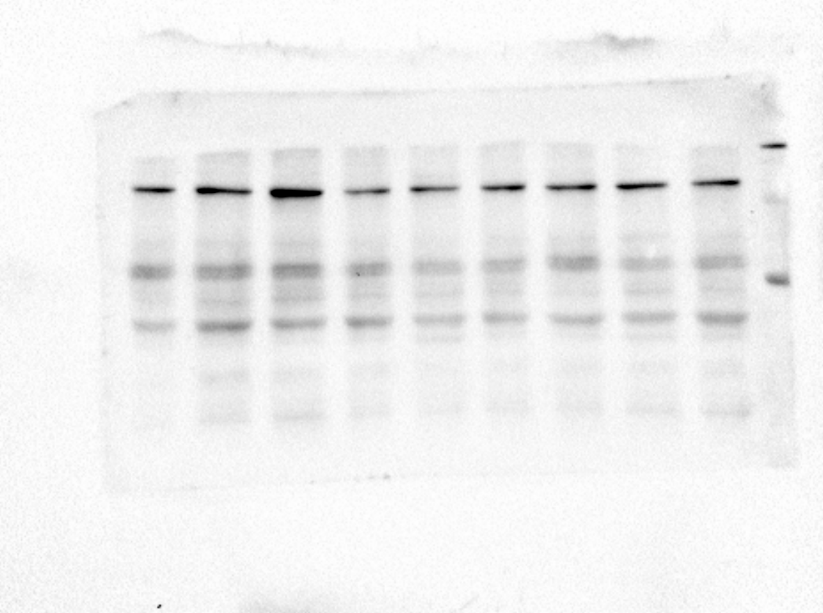
**
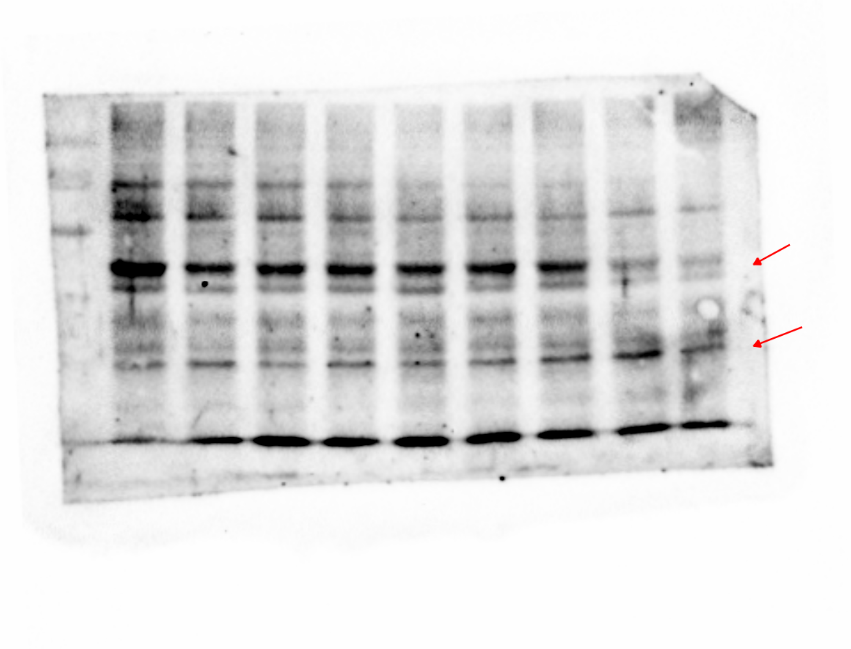


Stat3（88kDa）

IL-6（23kDa）

β-Actin（42kDa）

p-Stat3（88kDa）


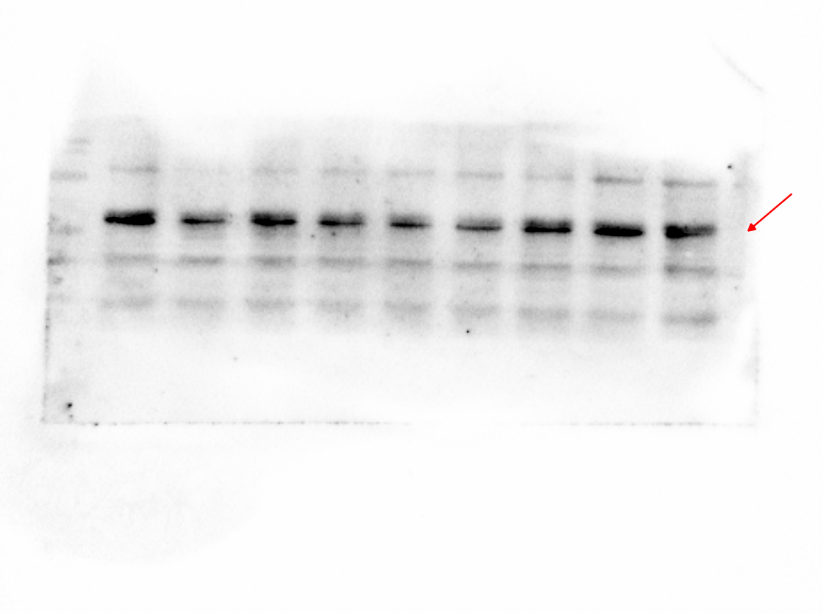


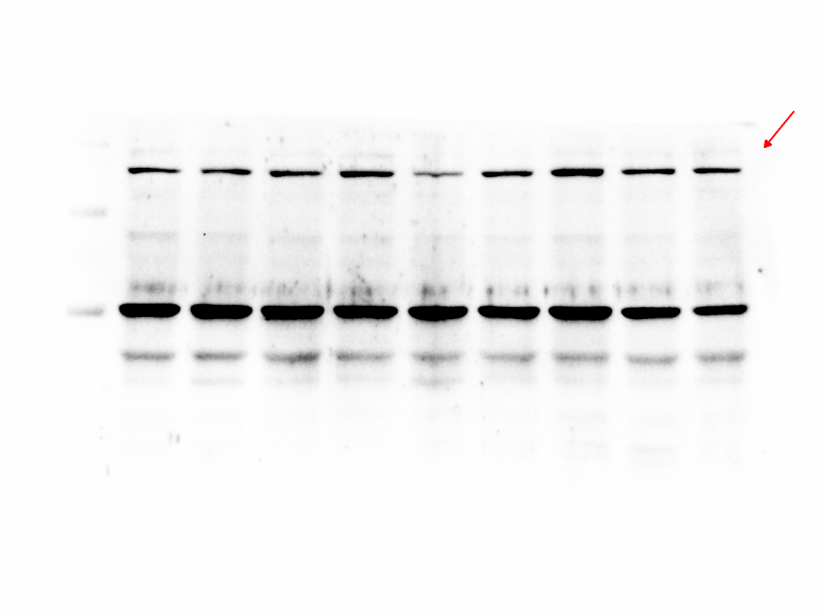

Supplement: Supplementary file 1 — Additional file 1: Figure 4E. Loading order: control group, TNBS group, mesalazine group, SYDH group, SYDM group, SYDL group. Figure 5A. Loading order in the red circle: control group, TNBS group, mesalazine group, SYDH group, SYDM group, SYDL group. [file 12906_2023_4237_MOESM1_ESM.docx]
